# Feasibility of a Mobile Health Tool for Mothers to Identify Neonatal Illness in Rural Uganda: Acceptability Study

**DOI:** 10.2196/16426

**Published:** 2020-02-18

**Authors:** Shababa B Matin, Allison Wallingford, Shicheng Xu, Natalie Ng, Anthony Ho, Madison Vanosdoll, Peter Waiswa, Alain B Labrique, Soumyadipta Acharya

**Affiliations:** 1 Center for Bioengineering Innovation and Design Department of Biomedical Engineering Johns Hopkins University Baltimore, MD United States; 2 School of Public Health Makerere University Kampala Uganda; 3 Bloomberg School of Public Health Johns Hopkins University Baltimore, MD United States; 4 Global mHealth Initiative Johns Hopkins University Baltimore, MD United States

**Keywords:** newborn, neonatal health, community health workers, maternal behavior, Uganda, World Health Organization, mobile apps, telemedicine

## Abstract

**Background:**

A shortage of community health workers to triage sick neonates and poor recognition of neonatal illness by mothers contribute significantly toward neonatal deaths in low- and middle-income countries. Providing low-resource communities with the tools and knowledge to recognize signs of neonatal distress can lead to early care-seeking behavior. To empower and educate mothers to recognize signs of neonatal illness, we developed a neonatal health assessment device consisting of a smartphone app and a wearable sensor (the NeMo system).

**Objective:**

The aim of this study was to determine if mothers in rural Uganda were willing and able to use the NeMo system during the first week of their infant’s life. We also assessed mothers’ responses to the device’s recommendation to seek care.

**Methods:**

A total of 20 mothers were enrolled in the study after giving birth in the Iganga District Hospital. Each mother was trained to use the NeMo system to assess her infant for signs of illness before leaving the hospital and was given the NeMo system to use at home for 1 week. Throughout the week, the smartphone tracked the mothers’ usage of NeMo, and the study team visited twice to observe mothers’ ability to use NeMo. Each mother was interviewed at the end of 1 week to gather qualitative feedback on her experience with the NeMo system.

**Results:**

In total, 18 mothers completed the study; 2 mothers were withdrawn during the week because of extenuating health circumstances. Moreover, 1 day after enrollment and training, 75% (15/20) of mothers used NeMo properly with no mistakes. Three days after enrollment and training, only 1 mother placed the wearable sensor improperly on her infant. On the final study day, only 1 mother connected the device improperly. Mothers used NeMo an average of 11.67 (SD 5.70) times on their own at home during the 5 full study days. Although the frequency of use per day decreased from day 1 to day 5 of the study (*P*=.04), 72% (13/18) of mothers used NeMo at least once per day. In total, 64% (9/14) of mothers who received an alert from the NeMo system to seek care for their infants either called the health care professional working with the study team or reused the system immediately and found no danger signs. All 18 mothers *agreed* or *strongly agreed* that the NeMo system was easy to use and helped them know when to seek care for their babies.

**Conclusions:**

NeMo is a feasible and acceptable tool to aid mothers in rural Uganda to assess their infant’s health.

## Introduction

### Background

An estimated 2.6 million neonatal deaths occur in the first month of life worldwide each year [1]. Roughly half of these deaths occur in the first 24 hours of life, with another 25% occurring in the first week of life [2,3]. Most of these deaths occur in low- and middle-income countries (LMICs) in community settings at home largely because of preventable causes such as sepsis, pneumonia, hypothermia, and complications of preterm birth, which could be prevented by timely identification of illness leading to early care-seeking behavior [2,4-9].

In Uganda, the neonatal mortality rate is estimated to be 27 per 1000 live births overall and 34 per 1000 in rural communities [10,11]. Like in other sub-Saharan African countries, a majority of neonatal deaths occur outside of the formal health system [12]. Poor recognition of neonatal illness is a major barrier for optimal care seeking by mothers [13]. An analysis of 64 neonatal deaths in eastern Uganda found that 54% occurred at home. Caretaker delay in problem recognition or in deciding to seek care was a major contributor to 50% of these deaths [14]. Many health care systems in LMICs, including Uganda, utilize village health teams (VHTs) of volunteer community health workers (CHWs) to visit mothers at home after they give birth to identify neonatal illness during the critical first week of life. In Bangladesh, neonatal assessment by a CHW has a sensitivity of 85% and a specificity of 75% in identifying infants 0-6 days old in need of admission to a facility [13]. Although CHW visits have been shown to lower the risk of neonatal death, only 5% of Ugandan neonates are visited in the first 48 hours of life because of the limited availability and bandwidth of CHWs [11,15].

Shifting the task of neonatal assessment and recognition of danger signs from CHWs to mothers is a promising strategy to improve early identification of neonatal illness [8,16]. However, the sensitivity of unassisted maternal recognition of symptoms of neonatal illness has been shown to be poor in developing countries [13,17,18]. Currently, the lack of knowledge and tools needed to evaluate neonatal health prevents families from identifying illness without CHW support.

The multidisciplinary team of students and faculty from the Johns Hopkins University Center for Bioengineering Innovation and Design (CBID), Johns Hopkins School of Public Health, and field partners from Makerere University in Uganda are developing an evidence-based system that we have named NeMo as a tool to guide mothers to identify danger signs of severe illness in their neonates. The goal of this system is to enable reliable and consistent assessment of neonates for identification of signs of illness to facilitate early referral of sick neonates, especially during the critical first week of life.

### The NeMo System

**Figure 1:**
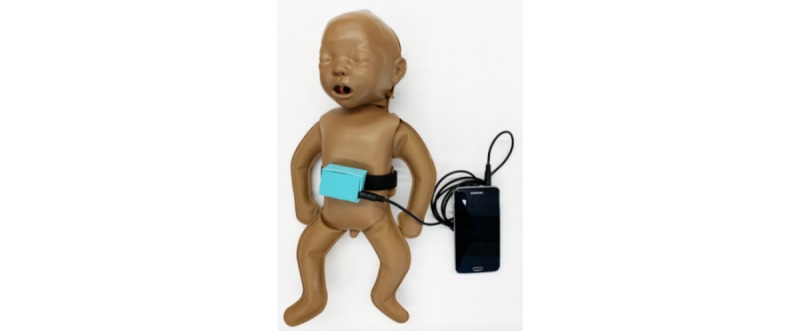
Smartphone connected by an audio cable to the NeMo band on a neonatal mannequin.

NeMo is a two-part system designed to empower mothers to identify the 4 qualitative symptoms most indicative of severe neonatal illness using a smartphone preloaded with an interactive app (the NeMo app) as well as detect respiratory distress using a low-cost, wearable sensing band (the NeMo band) that measures breathing rate (Figure 1). NeMo aids assessment of 5 danger signs of neonatal illness through the use of the app and band two to three times a day. The Integrated Management of Childhood Illness guidelines published by the World Health Organization promote a set of general danger signs to identify neonatal illness [19]. A subset of these clinical signs including difficulty feeding, convulsions, movement only when stimulated, respiratory rate of 60 breaths per minute or more, and severe chest indrawing have been previously identified as some of the most valuable predictors of a 0-59 day-old infant requiring hospital care [20].

**Figure 2:**
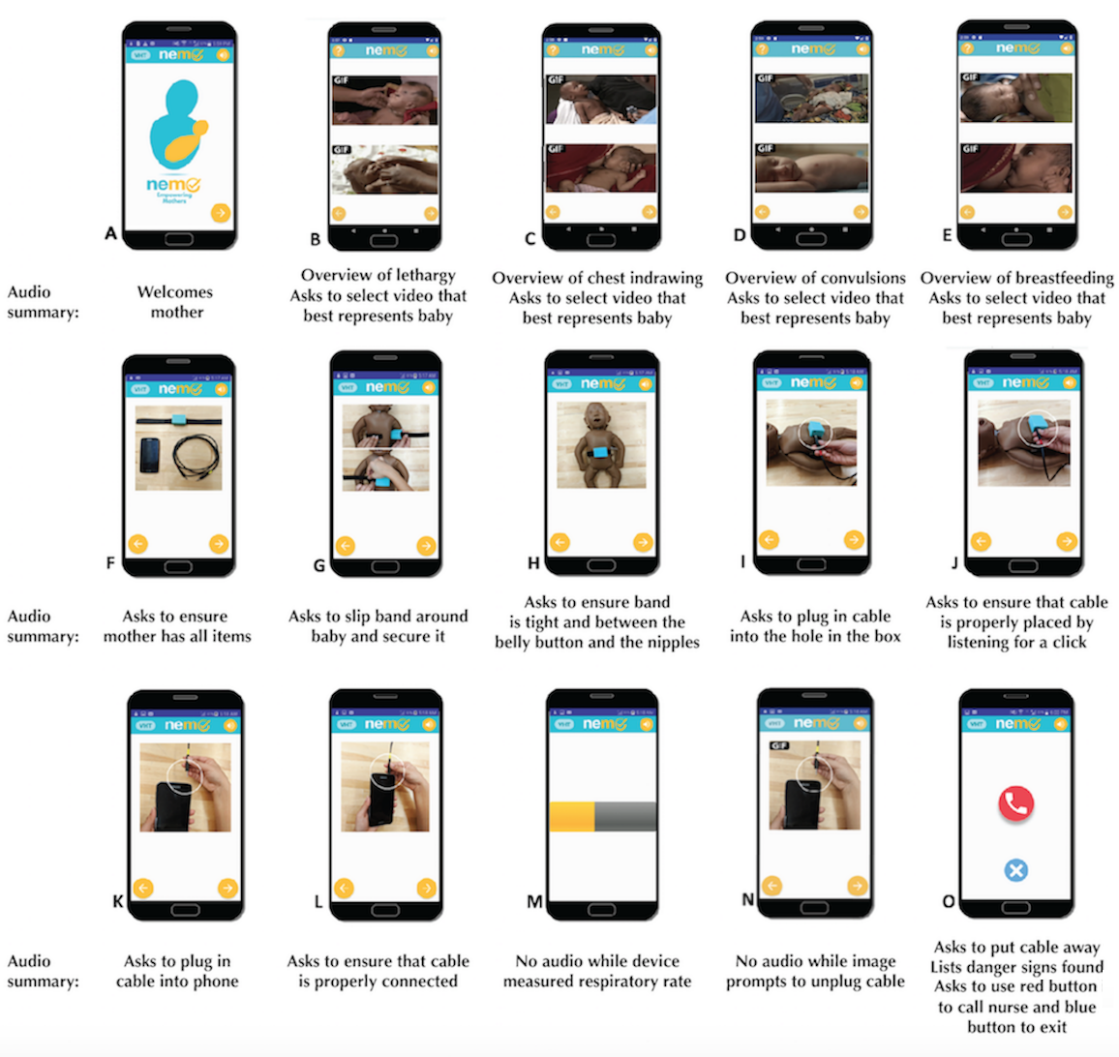
The user interface of the NeMo app.

Mothers are instructed to use the NeMo system thrice a day, for the first week of their baby’s life. The NeMo app interface has been previously described by Vanosdoll et al [21]. The app includes pictures, symbols, and audio recordings in the local language so that any mother, regardless of literacy, is able to navigate the app and examine her baby for signs of illness. The user interface of the NeMo app is shown in Figure 2. The steps are as follows: the initial home screen greets the mother and asks her to press the arrow to proceed with the assessment (Figure 2, A). The following 4 screens ask her to indicate the presence of the 4 qualitative danger signs in her infant: lethargy (Figure 2, B), chest indrawing (Figure 2, C), convulsions (Figure 2, D), and difficulty breastfeeding (Figure 2, E) by displaying 2 Graphic Interchange Format (GIF) images, one showing a newborn exhibiting the danger sign and one showing a healthy infant. The mother is asked to select the picture that best represents her newborn. Arrows, along with audio instruction, appear to direct the mother to move to the next or the previous screen once an image is selected. The following 7 screens use audio and visual instructions to guide the mother through properly placing the band around her baby and connecting the band and phone via the audio cord (Figure 2, F-L). The NeMo band connects to the smartphone through an audio cable via the headphone jack, which transmits signal for processing. The NeMo band uses a sensor housed in a plastic box with a silicone cover that is mounted on an elastic strap, the ends of which are attached using a generic hook-and-loop fastener similar to a Velcro closure. The sensor is fastened on top of the abdomen and detects abdominal movement to measure respiratory rate.

Upon completion of band placement, the NeMo band measures respiratory rate for 1 minute while showing the progress on the screen (Figure 2, M). Once the measurement is complete, a screen with a GIF demonstrating audio cord removal appears (Figure 2, N). As the speaker is inherently disabled when the phone detects an audio jack connection, no audio can be heard for this instruction. When the cable is removed, reenabling audio, the final screen instructs the mother to remove and store the device until the next use. The final screen of the app lists any danger signs detected and, if at least one danger sign is present, recommends that the mother seek medical attention for her baby. The final screen further recommends that the mother still seek care even if she believes her infant to be sick, but the device has not detected a danger sign. Two icons appear at the end of the audio recording. The mother can press the red phone icon to call a health care worker or press the blue X to close the app (Figure 2, O).

### Objectives

We undertook a small-scale pilot study of the NeMo system to determine the acceptability and ease of use of the system in the home setting. The primary objective of the study was to understand the willingness of mothers in rural Ugandan villages to use the investigational device in a real-world scenario. We also hoped to better understand barriers to adoption to inform future iteration on and implementation of the system.

The study imitated a real-world scenario of a mother using the device in her home to assess her infant’s health two to three times per day for the first week of life. The general research aim was to assess usage of the device by mothers after training to ascertain the NeMo system’s level of acceptability with mothers. Specific research aims included determining the following:

1. Mothers’ skill retention of correct device usage following an initial training session
2. Mothers’ willingness to use the device at home
3. Mothers’ initiative to seek care for their neonates based on NeMo’s recommendations.

## Methods

### Study Sites and Collaborators

This study was undertaken in eastern Uganda in the Iganga-Mayuge districts over the course of 3 weeks. The population demographic in these two districts is considered representative of rural Uganda with regard to socioeconomic landscape, education levels, and neonatal mortality rates [14,22,23]. The study team was accompanied by a team of local health workers including nursing officers and midwives based out of Iganga District Hospital, who also acted as translators for the study team. Study coordination was aided by Uganda Development and Health Associates (UDHA). The study was approved by the Johns Hopkins Institutional Review Board (IRB#00191743), the Institutional Review Board of Makerere University School of Public Health, and The AIDS Support Organization in Uganda. Written consent was obtained from all participants and was available in Lusoga and English. Before the start of the study, the NeMo device’s performance was validated in the Johns Hopkins Hospital nursery in a separate study (IRB#00117008) described elsewhere [24].

### Study Design

The study involved recruitment of mothers in the Iganga District Hospital, training participants to use the NeMo system before discharge from the hospital, and lending participants the device to use for 1 week at home. The study team visited participants' homes twice during the week.

### Participant Enrollment

Overall, 20 women who gave birth at Iganga District Hospital were enrolled before they were discharged from the hospital. The inclusion criteria of the study required that the participant must be aged 18-45 years, must be clinically stable, and must have a clinically stable infant under 7 days of age. The team selectively enrolled a diverse study cohort based on the following factors to best represent participants’ differing socioeconomic status: distance of participant’s residence from the hospital, number of antenatal care sessions attended, and cellphone access. Participants were told during recruitment that they would receive a small gift at the end of the study as a token of appreciation, with no mention of its value.

### Training to Use the NeMo System

Participants were interviewed to obtain demographic information using a structured interview guide and trained to use the NeMo system before they were discharged from the hospital. Participants were first asked to identify sick children from a series of videos to assess baseline knowledge of illness, after which they received a short lesson on neonatal danger signs. Participants were then taught to use the NeMo system by answering the danger sign questions on the NeMo app when shown additional videos of sick and healthy children. Participants practiced placing the band on a neonatal mannequin and, finally, completed a full use of the system on their own babies. A detailed description of the training protocol can be found in Multimedia Appendix 1.

### Device Usage in the Home

**Figure 3:**
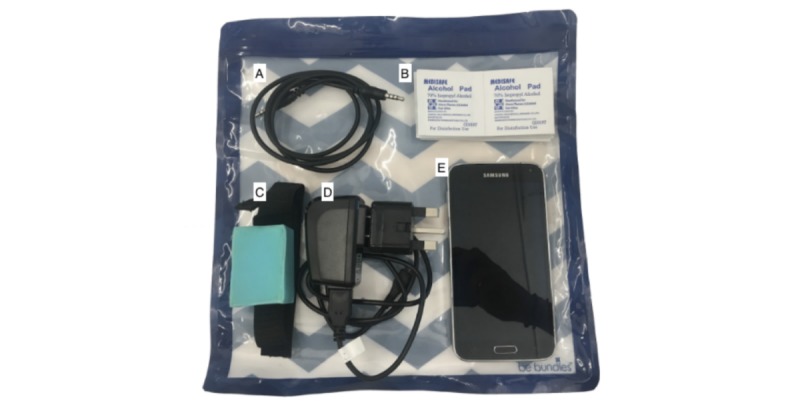
Equipment given to each study participant for the duration of the study: (A) 3.5-mm male-to-male TRRS audio cable, (B) Alcohol wipes, (C) NeMo device, (D) Smartphone charging cable with type-G adapter, and (E) Samsung Galaxy S5 smartphone.

Upon completion of training, mothers were provided with a heavy-duty zip bag, as shown in Figure 3. The smartphone was loaded with the NeMo app, and all other apps were hidden to prevent confusion and improper use of the phone. Mothers were instructed to use NeMo three times each day (morning, noon, and night) to assess their baby’s health daily during the first week of the baby’s life. The study team members collected the zip bag and its contents at the end of the week. Each mother was provided an unused NeMo device to decrease the risk of cross-contamination between neonates.

**Figure 4:**
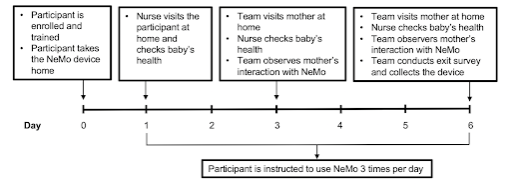
Study protocol for each day of the study beginning with enrollment on day 0 and ending with exit on day 6.

A member of the health worker team visited the mothers in their homes on study day 1 to assess each baby’s health and to observe the mothers using the device, address concerns, and correct any improper usage. The study team, accompanied by a health worker, visited mothers in their homes on study days 3 and 6 to observe device usage, conduct interviews, and assess each baby’s health, as summarized in Figure 4. This framework was designed to mimic the recommended CHW postbirth visitation schedule. At no point during the visits were the mothers asked to use the device more frequently. Mothers received a small gift consisting of baby items, costing a total of 30,000 UGX (US $8) at the end of the study.

### Data Collection

During training, data on baseline assessment of danger sign knowledge and rounds of training video vignettes were collected. The study team collected qualitative feedback and responses from mothers using a Likert scale questionnaire and structured interview guides during each encounter, to assess insights related to device use.

During home visits, study team members observed device usage by the mother and recorded any mistakes. Incorrect use was immediately corrected following observation. During visits on days 3 and 6, a video recording was taken of the mother using the device for future reference.

The backend of the NeMo app recorded the mother’s responses to the qualitative danger sign questions and the respiratory rate measured throughout the study period. Each screen opening and button press was time-stamped during each use. The mother was considered to have successfully completed a device use if the phone recorded an interaction with every single screen within the app.

### Data Analysis

#### Training and Skill Retention

The average score on the danger signs baseline assessment was tested for significance using a *t* test. The null hypothesis of this *t* test was that mothers would score an average of 8/16 correct, as this is the most probable score through random guessing. One-way analysis of variance (ANOVA) was performed to detect significant differences in scores between rounds of video vignettes in phase 3 of training (Multimedia Appendix 1). Skill retention was determined as correct steps of use completed on observation.

#### Frequency of Use and Impressions of Device

Quantitative data, including mothers’ number of uses tracked by the phone, time spent on each screen, and Likert scale responses, were analyzed by manual sorting and summarizing. Repeated measures ANOVA was used to detect significant differences in device use frequency between study days, and posthoc Tukey tests were conducted to detect where those differences occurred. Qualitative data including the study team’s observations about usage and mothers’ interview responses were decoded and de-identified and then analyzed by employing themes identified a priori, including opportunities and barriers surrounding the NeMo system within the homes of new mothers.

#### Responses to Danger Sign Trigger

Danger sign triggers and the corresponding responses by mothers were summarized and categorized. Mothers’ responses were analyzed through a qualitative discussion.

## Results

### Study Population Demographics

The study cohort consisted of mothers from 19 villages across the Iganga-Mayuge districts; distances between the mothers’ homes and Iganga District Hospital stretched from 400 m to 24 km. During the 3-week study period, 20 mothers were recruited and 18 completed the study. Moreover, 2 participants were withdrawn in the course of the study because of extenuating health circumstances. Data collected from these 2 participants before they were withdrawn from the study, including day 0 interview responses, training results, and device uses, are included in these results. Background information from participants is shown in Table 1.

**Table 1:** Demographics of women enrolled in the study (N=20).

Characteristic		Values	
Age (years), mean (SD)		27.2 (6.84)	
Distance from hospital (km), mean (range)		7.78 (0.40-24)	
Parity, mean (range)		3.5 (1-7)	
First time mothers, n (%)		5 (25)	
Own a phone, n (%)		11 (55)	
Have ever used a smartphone, n (%)		8 (40)	
Able to charge a phone, n (%)		18 (90)	
Attended ANC^a^, n (%)		19 (95)	
ANC sessions attended, mean (range)		4 (0-5)	
**Knew danger sign, n (%)**			
	Failure to breastfeed		8 (40)
	Convulsions		5 (25)
	Breathing difficulty		2 (10)
	Lethargy		2 (10)

^a^ANC: antenatal care.

### Training Results

Training sessions were successfully completed by each of the study participants and took an average of 1.5-2.5 hours to complete. During the initial preassessment of baseline knowledge of neonatal illness (Multimedia Appendix 1), the average score on the initial danger sign knowledge assessment was 13.95 (SD 0.94) out of 16, which was significantly higher than random guessing (*P*<.001). However, 55% (11/20) of mothers were unable to list the correct presentation of illness despite correctly identifying that the baby in the video was sick. One-way ANOVA did not reveal a significant difference in mothers’ average scores between the first, second, and third rounds (*P*=.36) of practicing danger sign identification using the NeMo app.

Table 2 summarizes correct task completion when the mother used NeMo for the first time on her baby during training on day 0. The two largest sources of error were incorrect placement of the band and failure to disconnect the audio cord from the phone at the end of the respiratory rate and temperature assessment. The largest contributing factor to incorrect band placement was failure to appropriately tighten the band around the baby’s abdomen.

All mothers indicated that the voice directions in the app were understandable, and 75% (15/20) of the mothers indicated that they had learned at least one new neonatal danger sign. Following training, 100% (20/20) of mothers stated that they would feel comfortable using the device three times per day. During the study exit on the last day of the study, 83% (15/18) of mothers indicated that the training protocol they underwent on day 0 was sufficient to enable independent use of the device in their homes.

**Table 2:** Percentage of mothers who correctly completed each task during the observed uses on neonates.

Task	Mothers who correctly completed each task, n (%)			
	Day 0 (training)	Day 1	Day 3	Day 6
Unlocked the phone and opened the app	20 (100)	20 (100)	18 (100)	18 (100)
Navigated properly through the app	17 (85)	14 (70)	18 (100)	18 (100)
Correctly answered qualitative questions	20 (100)	20 (100)	18 (100)	18 (100)
Properly placed the device on the neonate’s abdomen	6 (30)	18 (90)	17 (94)	18 (100)
Properly connected the audio cord to both audio jacks	16 (80)	20 (100)	18 (100)	17 (94)
Disconnected the audio cord from phone unprompted	7 (35)	18 (90)	18 (100)	18 (100)
Closed the app to acknowledge assessment finished	19 (95)	19 (95)	18 (100)	18 (100)

### Skill Retention

During the day 1 visit, 75% (15/20) of mothers were able to properly execute all the steps of using the device on their infants in front of the health worker. The remaining 25% (5/20) of mothers were retrained by the visiting health worker. Mistakes with device placement included placing the device too loosely to obtain respiratory rate (1 mother), placing the box upside down on the abdomen (1 mother), and failing to unplug the connection cord from the phone without prompting (2 mothers). In addition, 3 mothers navigated the app improperly, which resulted in skipping the audio indicating if any danger signs had been detected. During the day 3 visit, only 5% (1/18) of mothers needed to be retrained, as they placed the sensor box upside down. Only 1 mother needed to be prompted to unplug the connection cord when observed on the day 6 visit.

### Device Usage by Mothers

**Figure 5:**
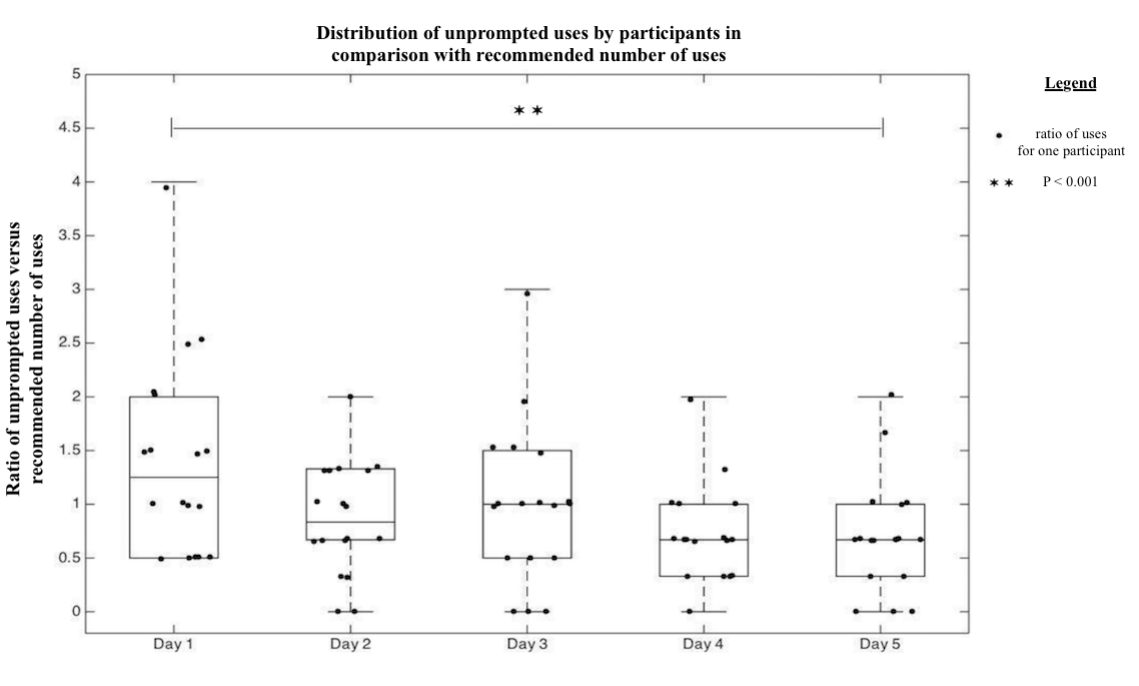
Box and whiskers plot of normalized unprompted uses expressed as a ratio of unprompted uses and expected number of uses. For example, on day 1, mothers on average used NeMo 1.25 times more than expected. Each point represents the ratio of uses by one participant on a corresponding day. Expected number of unprompted uses was 2 times on days 1 and 3 and 3 times on days 2, 4, and 5.

The 20 mothers enrolled in the study successfully completed the app a total of 252 times over the course of the study. On day 1 and day 3, the mother was asked to use the device while the study team observed, and this use was counted as a *prompted use*. The uses that the mother performed by herself at home, which were recorded by the phone, were counted as *unprompted uses*. On average, mothers performed 11.67 (SD 5.70) complete, unprompted uses per week-long study period. As the mother was prompted to use the device once on day 1 and day 3, we considered the ideal number of unprompted uses on these days to be two uses, compared with the ideal number of three unprompted uses on the other days. The difference between actual uses (mean 11.67, SD 5.70) compared with the ideal 13 times is not statistically significant (one-sample *t* test, *P*=.33). The frequency of use for each day has been normalized on a ratio of the ideal use of that day and presented in Figure 5 as the distribution of complete, unprompted uses by mothers on each day of the study.

**Figure 6:**
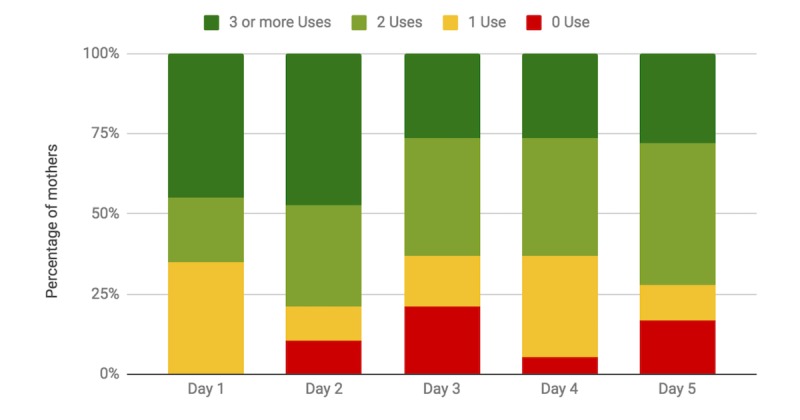
Distribution of the percentage of mothers who used the device unprompted 0 times, 1 time, 2 times, and 3 times or more, over the course of 5 days. Most mothers used NeMo 2 or more times a day, and very few mothers failed to use the device on any given day.

There was a significant difference between the normalized frequency of unprompted use across the days of the study (repeated measures ANOVA, *P*=.005). A significant drop in normalized frequency was found between day 1 and day 7 of the study (posthoc Tukey test, *P*=.04); however, a significant difference was not found between any other 2 days. Figure 6 shows the distribution of the percentage of mothers who completed a device use 0, 1, 2, and 3 or more times each day.

Of the 18 participants who completed the study, 13 (72%) used NeMo consistently, completing a device use at least once per day. Only 2 participants skipped using NeMo for 2 or more consecutive days. In all, 9 mothers used the device more than the recommended three times a day at least once. From the exit survey, reasons for not using NeMo three times a day included social obligations outside the home, refusal to unwrap the baby on cold mornings, housework, caring for a sick child, and the phone being sent away for charging.

Mothers consistently used the device on their own at home, with most mothers using it at least once every day. Half of the mothers stated that they would like to use the system even more frequently than recommended because it helped them to monitor the health of their baby.

### Neonatal Illness Assessment by Mothers at Home

**Figure 7:**
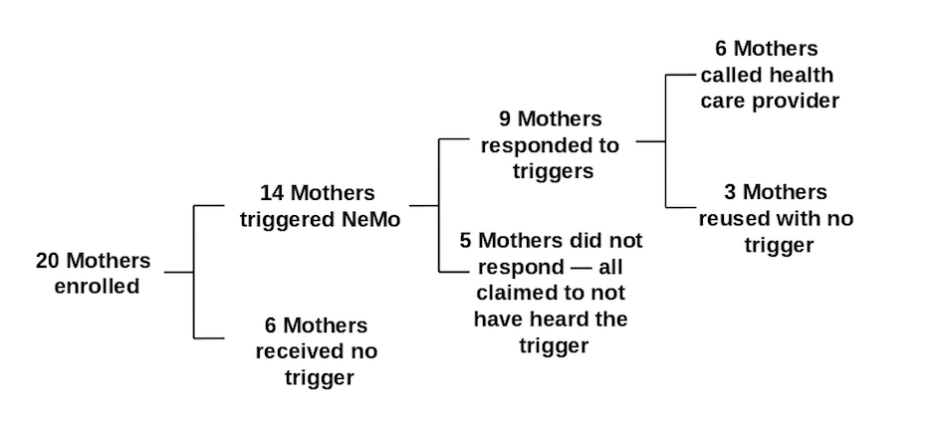
Summary of responses to NeMo danger sign triggers.

In total, 14 mothers received a total of 63 triggers from the NeMo app warning them that their baby had an elevated respiratory rate; 5 of these mothers did not recall hearing the warning from the phone when asked about it during their follow-up interviews. None of these 5 babies were actually sick when assessed by the study nurse. In total, 9 mothers indicated that they were aware of a trigger. We considered the mother to have responded to the trigger if she either called the health care provider or reused NeMo on her baby to find that the baby was healthy. Figure 7 shows a summary of responses to triggers.

In total, 6 mothers contacted a health care provider following at least one of the triggers they received. One of the babies of a mother who called had a cord infection and was brought to Iganga District Hospital. In addition, another baby whose mother neither received a trigger from NeMo nor called was found during the day 1 visit to have a mild cord infection and was treated at home with medication and proper cord care. Another mother called the on-call health care professional out of concern for her baby’s health without prompting from NeMo.

### Impressions of Device

**Figure 8:**
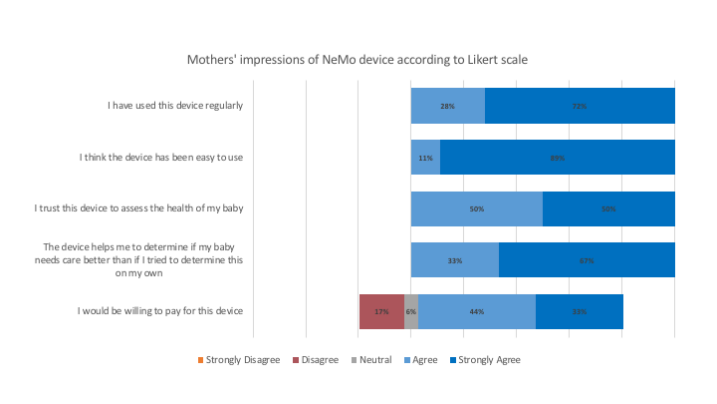
Mothers’ responses to Likert scale statements regarding their experience using the device after 6 days.

During the exit interview, each participant was asked about her experience with and impressions of the NeMo system using a Likert scale. Mothers’ responses to each statement are shown in Figure 8.

### Willingness to Use

Using a composite scoring system (strongly agree=5 and strongly disagree=1), the statement “I have used the device regularly” had a composite score of 4.72 (median 5, IQR 1). The statement “I would trust this device to assess the health of my baby” had a composite score of 4.5 (median 4.5, IQR 1). All 18 mothers who completed the study answered that they would like to use the device again if they had another baby, and 94% (17/18) of mothers said that they would recommend using the device to other women in their village. During the exit survey, 5 mothers stated that they thought the device should be used more than three times a day and suggested instead that recommended daily usage be increased to four or five times.

Throughout the study, mothers were encouraged to express their concerns and ask questions about the device. Few mothers expressed concerns about using the device. Common themes include concerns about the electrical safety of the device, potential stress to the baby caused by the band, and unwillingness to unwrap the baby to put the device on when the weather is cold. All of the reported concerns or questions were answered by the team when they were raised.

### Ease of Use

All mothers *agree* or *strongly agree* that the device was easy to use, giving a composite score of 4.89 (median 5, IQR 0). All 18 mothers answered that they had become more comfortable using the device over the course of the study.

### Perception of Usefulness

All mothers *agree* or *strongly agree* that the device helped them know when to seek care for their babies, resulting in a composite score of 4.66 (median 5, IQR 0). The most common reasons given for agreeing with this statement were that the device can assess breathing rate, which the mother cannot do on her own (4 mothers), and the device displays the danger signs (3 mothers). In total, 89% (16/18) of the participants who completed the exit survey said that other mothers would find the device useful.

### Device Maintenance

Out of the 20 enrolled mothers, 11 (55%) were able to charge phones at home, whereas the rest needed to charge at a neighbor’s house or nearby charging station up to 1.5 km from their home. During recruitment, 10% (2/20) of the mothers answered that they would have major difficulties (ie, have to travel a long distance or pay) and were, thus, provided with power banks to facilitate charging. Out of the 18 participants who completed the study, 11 (61%) charged the phone at least once during the study period. There were no adverse system events (including device breakage or loss of either phone or device) during the study despite 55% (10/18) of participants expressing concerns about losing or breaking the device. All devices remained intact and functional by day 6 when the devices were collected.

## Discussion

### Principal Findings

In this study, we assessed if mothers in rural Uganda were willing and able to use the NeMo system during the first week of their infant’s life and if NeMo would trigger care-seeking behavior. Findings confirmed that mothers in rural Uganda would benefit from more education on recognizing signs of neonatal illness. The skill retention and usage results demonstrated that NeMo has a high level of usability and acceptability among mothers. Usability improvements could potentially increase the accuracy of the sensor and users’ understanding of care-seeking recommendations.

### Training and Skill Retention

Enrolled mothers could recognize a child in clear, visible distress as sick with significantly better accuracy than random guessing. However, most mothers were unable to list a specific reason for illness, which is in line with previous studies demonstrating poor knowledge of danger signs among women in community settings. Although mothers scored well when identifying very pronounced examples of neonatal distress shown during the training, education of specific danger signs is needed to enable early recognition of more subtle presentations of illness.

Mothers’ ability to correctly use NeMo increased over the week through repeated use, showing that the overall system is intuitive to use. In this study, it was found that only the inability to consistently tighten and place the band arose as a primary usability concern. To obtain a signal, the sensor must be placed centrally on the neonate’s abdomen (in between the nipples and midway between the umbilical cord and the nipples) and the band must be secured tightly around the baby, such that the outward movement of the baby’s abdomen during a breath presses on the underside of the box (see Figure 1). Throughout the study, the team observed that erroneous respiratory rate readings resulted from incorrect band placements that were too loose or too tight, band placement on the chest rather than the abdomen, and poor audio jack connection to the device. In addition to a better training protocol, which better addresses band placement, other usability improvements such as reengineering of the band to eliminate memory burden for mothers must be made before any large-scale implementation efforts.

### Willingness to Use

For NeMo to be an effective task-shifting tool, it must be well accepted by mothers such that they are willing to use the device regularly to assess their babies.

Mothers enrolled in our study consistently used the device on their own at home, with most mothers using it at least once every day. The average use at each day was at least two times. The study recommended mothers to use NeMo three times a day to ensure that enrolled babies were screened as often as possible without overburdening mothers. However, the use of NeMo even once a day greatly raises the frequency of neonatal screening during the first week of life. With 72% (13/18) of mothers using NeMo at least once every day, and the weekly average number of uses being 11.67 (SD 5.70), enrolled babies in the first week of life were being assessed approximately 10 times more with NeMo than they would have been within the current health care system in Uganda, where VHTs are overburdened and, thus, unavailable to carry out neonatal assessment with the recommended frequency. Therefore, NeMo has the potential to address this gap in the health care system.

A large number of mothers used the device more than the recommended three times on a given day of the study, especially on the first study day (Figure 5). The increased frequency of usage could be because of the novelty factor of NeMo among mothers, which must be considered in interpreting the generalizability of the usage results. Longer-term, large-scale studies are needed to assess the frequency of usage among mothers in a community that has become familiar with the NeMo device. Usage in excess of three times per day may also reflect mothers’ heightened anxiety about the health of their babies, especially in the first days of life, which may translate to frequent NeMo usage in a real-use case.

### Behavior Change

NeMo’s ability to task-shift neonatal assessment to mothers to reduce preventable neonatal deaths depends on its ability to initiate care-seeking behavior in mothers. Mothers’ responses to its recommendations to seek care serve as indicators of the behavior change initiated by NeMo. The cases observed suggest that mothers who claimed to understand the device’s audio recommendation to seek care are led to take further action to protect the baby’s health.

During the study, the device signaled false-positive triggers for 13 mothers and one true-positive trigger for a sick neonate. Our observations suggest that many false positives were likely because of erroneous placement of the band. For all mothers who received a high respiratory rate trigger in the presence of the study team, it was observed that upon tightening of the band around the neonate, a normal respiratory rate was then recorded. Therefore, future iterations of the device require further considerations in human factors design that mitigate incorrect band placement and, thus, false-positive respiratory rate findings.

Out of the 14 mothers who received a trigger from the device, 5 claimed to have not heard the recommendation to seek care. Our posthoc analysis suggests that the app’s audio and visual prompts to indicate the presence of a danger sign may have been unclear or too subtle, which may be responsible for some of these failures to acknowledge care-seeking recommendations. Modification of the app user interface to make the danger sign trigger more obvious may increase understanding of the danger sign trigger.

The remaining 64% (9/14) of mothers who received a trigger took action to further assess the baby’s health, which the team interpreted as understanding and trusting the trigger. The team considers the mothers who used the device again immediately after the trigger to find no danger signs to have had an appropriate response to the trigger. The behavior of the 6 mothers who called the health care worker immediately following a trigger indicates NeMo’s potential to lead mothers to seek care for sick newborns.

A major risk of the NeMo device is false negatives wherein the device would fail to detect a danger sign in a sick newborn, resulting in a mother not seeking care because of false reassurance from the device. The behavior of mothers in our small cohort does not show evidence that the device will deter mothers of sick infants from seeking care, although the study was not powered to detect such events; 1 mother even called the health care professional without prompting by the NeMo device.

### Limitations of Study

This acceptability study gained preliminary evidence on the feasibility of NeMo implementation. Given the formative nature of the study, it was not powered to show effectiveness of the intervention. Rather, the objective of this study was to show proof-of-concept and to identify any major barriers to implementation so that a more rigorous randomized controlled trial (RCT) could be carried out based on the results.

The study was not able to assess the ability of mothers to identify qualitative danger signs aided by the NeMo app, as none of the babies of mothers enrolled displayed any of these danger signs, and none of the mothers answered in the NeMo app that her baby had a danger sign.

Mothers may have been influenced to use the device more or less frequently because of the intermittent visits by the health care provider and the study team. On one hand, mothers may have been reminded or encouraged to use the device by the presence of the study team. Conversely, the mothers may have been discouraged from assessing their infants on their own because the infant’s health was assessed during the visit. The visits were limited to the days that a CHW would be expected to visit to simulate the reminders to use the device that a mother might be expected to receive in a real-use case. However, CHWs rarely visit as often as the recommended three times in the first week of life.

As a result of the sample size and the recruitment protocol, the generalizability of the study was limited. For example, being able to charge the smartphone was not among the selection criteria, and power banks were given to 2 mothers who expressed difficulty charging phones at home. Therefore, the willingness of mothers with difficulty accessing the power grid to go out of their way to use the device could not be determined.

This study was limited in its ability to assess the potential of the NeMo device to initiate care-seeking behavior in a real-use case in the local health care system. Mothers in the study who wished to seek care for their infants were provided with access to an on-call health care worker, rather than make a costly and arduous trip to a health center. We provided an on-call health care worker for the ethical consideration that using the NeMo device may burden mothers unnecessarily in the cases of false positives or may deter mothers from seeking care in the cases of false negatives. Studies have shown significant financial and sociocultural barriers to seeking medical care in the communities the NeMo device targets, such as financial constraints, distance from medical facilities, and reliance on traditional medicine [25].

### Future Directions for the NeMo System

In light of the findings from this study, the next steps for the NeMo system include reengineering of the band to reduce the memory burden to mothers, reducing the occurrence of false-positive danger sign triggers and making it obvious to mothers that danger signs have been found in her baby. To facilitate implementation to scale, models that make smartphones accessible must be considered.

To accurately measure quantitative danger signs and prevent false-positive triggers, the user needs to place the NeMo band at the correct location and with appropriate tightness. Most mothers could perform the former task with the help of audio and visual directions but struggled with the latter. To guide mothers to place the band consistently and correctly, a visual indicator on the band that reflects the tightness could be added. In addition, other visual indicators such as arrows indicating orientation of placement may also increase accuracy of device placement. To draw the mother’s attention to any danger sign triggers, visual cues as well as audio alarms that indicate the appropriate danger signs may be added.

The requirement of a smartphone remains the biggest barrier to widespread implementation of NeMo. For implementation to scale, various business and public health models need to be investigated to make smartphones accessible to those who do not own one. Multiple models are currently being investigated by the research team for this purpose.

The larger implication of the implementation of a mobile health system like NeMo is the increased referral of neonates demonstrating danger signs. The NeMo system only addresses the creation of early care-seeking behavior. To save neonatal lives, a responsive supply chain of treatment must also be established.

### Conclusions

The study presented here demonstrated acceptability and feasibility of mothers in a rural community using a NeMo device on their own at home. Mothers in rural eastern Uganda were willing to use the NeMo system to assess their infants frequently during the first week of life in a simulated use case. Mothers were able to understand how to use the NeMo system with minimal training and retained the skill to use the device for 6 days. Most importantly, the 9 mothers who claimed to have understood the device’s trigger to seek care took steps to ensure the health of their infants, demonstrating the ability of the NeMo system to initiate early care seeking by mothers. These findings indicate mothers’ increased awareness and understanding of neonatal illness, which suggests that NeMo could contribute to more referrals leading to higher neonatal survival rates. The results of this study also provide insights that will guide improvements in future prototypes of the NeMo system, for example, the next iterations of the NeMo system will incorporate a clearer danger sign trigger and assessment of an expanded set of danger signs (temperature and cord infection).

Implementation of the NeMo system has the potential to become a public health intervention by empowering and educating mothers to identify neonatal illness to promote early care-seeking behavior. Following further prototype iteration, the NeMo system will be introduced in the community in a large-scale RCT to validate the use of this intervention to reduce neonatal deaths in the home.

## Appendix

Multimedia Appendix 1 Training protocol.

## Abbreviations

ANOVA: analysis of variance
CBID: Center for Bioengineering Innovation and Design
CHW: community health worker
GIF: Graphic Interchange Format
LMIC: low- and middle-income countries
RCT: randomized controlled trial
UDHA: Uganda Development and Health Associates
VHT: village health team
