# Supplementary material for: Feasibility of a Mobile Health Tool for Mothers to Identify Neonatal Illness in Rural Uganda: Acceptability Study
Source: JMIR Mhealth Uhealth. 2020 Feb 18;8(2):e16426. doi: 10.2196/16426 (PMC7055749; doi:10.2196/16426)
Supplement: Multimedia Appendix 1 [file mhealth_v8i2e16426_app1.docx]

## Appendix A: Training Protocol

Participants were trained in five phases: (1) pre-assessment of knowledge, (2) danger sign training, (3) phone operation, app instruction, & video vignette post-assessment, (4) NeoNatalie^TM^ band placement, and (5) newborn band placement.

### Phase 1: Pre-assessment of Knowledge

To assess mothers’ ability to identify the four qualitative danger signs screened for by the NeMo system (lethargy, chest indrawing, convulsions, failure to breastfeed), mothers were shown 16 video vignettes of either healthy or sick newborns demonstrating one of the four relevant danger signs. They were asked to indicate their interpretation of the newborn’s condition in each video.

### Phase 2: Danger Sign Training

Using GIF examples presented on a tablet, participants were given a short lesson on how to identify the four qualitative danger signs in newborns.

### Phase 3: Phone Operation, App Instruction, & Video Vignettes

Participants were trained in the basics of smartphone operation, including how to unlock the phone, charge the phone, adjust the volume, and open the NeMo app. A study team member explained to the participant the premise of the app and guided them through app navigation.

The mother then was asked to practice answering the four qualitative danger sign questions (lethargy, chest indrawing, convulsions, failure to breastfeed) posed by the app (Figure 2B-E). Each time the mother used the app she was shown one video vignette round consisting of four GIFs pertaining to each of the qualitative signs. Each GIF depicted either a healthy infant or an infant afflicted with a relevant danger sign. The mother answered in the app whether she believed the infant in the GIF had the danger sign. A total of five rounds of video vignettes were created by the study team, and the rounds shown to each mother was selected using a random numerical sequence generator tool. Mothers underwent a minimum of three rounds of video vignettes and completed up to two additional rounds of video vignettes if they continued to struggle with app navigation or danger sign identification.

### Phase 4: NeoNatalie^TM^ Band Placement

Mothers were briefly instructed on how to place the band on a NeoNatalie^TM^ (Laerdal) infant mannequin by a study team member. They were then asked to follow the instructional section of the NeMo app to practice placing the band on the mannequin and connecting the device to the smartphone with the audio cable (Figure 2F-L). Study team members explained the last screen of the app, which warns of any danger signs identified. Mothers were instructed to check and adjust the tightness of the band if NeMo detected fast breathing. They were then asked to wait a few minutes before reusing the NeMo system a second time and call a nurse if the system detected fast breathing a second time.

### Phase 5: Newborn Band Placement

Mothers then performed a complete use of the NeMo system on their newborns, answering the qualitative questions about their infants and placing the band to get a respiratory rate measurement and recommendation. Mothers who made mistakes during device use on their neonates were corrected. To reduce the risk of infection, mothers were instructed to wipe down the device with an alcohol wipe before each placement.
